# Design of a framework for the deployment of collaborative independent rare disease-centric registries: Gaucher disease registry model

**DOI:** 10.1016/j.bcmd.2017.01.013

**Published:** 2018-02

**Authors:** Matthew I. Bellgard, Kathryn R. Napier, Alan H. Bittles, Jeffrey Szer, Sue Fletcher, Nikolajs Zeps, Adam A. Hunter, Jack Goldblatt

**Affiliations:** a Centre for Comparative Genomics, Murdoch University, Murdoch, Western Australia, Australia; b Western Australian Neuroscience Research Institute, Nedlands, Western Australia, Australia; c Convenor of the Australian Bioinformatics Facility, Bioplatforms Australia, Macquarie University, North Ryde, New South Wales, Australia; d School of Medical and Health Sciences, Edith Cowan University, Joondalup, Western Australia, Australia; e Clinical Haematology and Bone Marrow Transplant Service, Royal Melbourne Hospital, Parkville, Victoria, Australia; f Genetic Services & Familial Cancer Program of Western Australia, King Edward Memorial Hospital, Subiaco, Western Australia, Australia

**Keywords:** IRDR, Independent Rare Disease Registries, RDRF, Rare Disease Registry Framework, GR, Gaucher registry, ICGG, International Collaborative Gaucher Group, DEs, data elements, RML, Registry Mark-up Language, RML, Registry Mark-up Language, API, application programming interface, Gaucher disease, Independent Rare Disease Registry, Open source, Post-market surveillance, Rare Disease Registry Framework, Web-based

## Abstract

Orphan drug clinical trials often are adversely affected by a lack of high quality treatment efficacy data that can be reliably compared across large patient cohorts derived from multiple governmental and country jurisdictions. It is critical that these patient data be captured with limited corporate involvement. For some time, there have been calls to develop collaborative, non-proprietary, patient-centric registries for post-market surveillance of aspects related to orphan drug efficacy. There is an urgent need for the development and sustainable deployment of these ‘independent’ registries that can capture comprehensive clinical, genetic and therapeutic information on patients with rare diseases. We therefore extended an open-source registry platform, the Rare Disease Registry Framework (RDRF) to establish an Independent Rare Disease Registry (IRDR). We engaged with an established rare disease community for Gaucher disease to determine system requirements, methods of data capture, consent, and reporting. A non-proprietary IRDR model is presented that can serve as autonomous data repository, but more importantly ensures that the relevant data can be made available to appropriate stakeholders in a secure, timely and efficient manner to improve clinical decision-making and the lives of those with a rare disease.

## 1 Introduction

Orphan drugs are those that are developed to treat specific rare conditions. However, clinical trials for orphan drugs frequently are complicated by a paucity of available patients, heterogeneity in onset, clinical course and genetic basis, and the scarcity of biomarkers to monitor response to therapy. Therapeutic dilemmas, such as the optimal age to start therapy and dosage at various stages of therapy, therefore, are often unresolved prior to a drug receiving marketing approval. Although regulatory authorities request post-marketing data, this information is generally only related to safety and is generated by the sponsoring drug company. Government incentives in the US, Australia and the EU for orphan drug development have resulted in significant advancement in the treatments of rare conditions [1]. But, treatment effectiveness across patient populations has become a contentious issue, with calls for reform of the post-authorisation assessment of orphan drugs through adaptive licensing processes [2]. One critical challenge is to acquire high quality treatment efficacy data that can be compared reliably across patient cohorts, and for rare conditions, typically derived across governmental and country jurisdictions. Most importantly, these patient data must be captured with limited corporate interference. As a result, there has been a call to develop collaborative, industry-independent, patient-centric registries for post-market surveillance of aspects related to drug efficacy [2].

The development and sustainable deployment of non-proprietary Independent Rare Disease Registries (IRDR) will serve as autonomous data repositories for the collection of comprehensive clinical, genetic and therapeutic information about patients with rare diseases. A resource of this nature has been identified as one of the “key outcomes from stakeholder workshops at a symposium to inform the development of an Australian national plan for rare diseases” [3].

In late 2014 the Wellcome Trust funded a Pathfinder Award to support and drive the establishment of an IRDR to serve as a model data source for the collection of comprehensive clinical and genetic rare disease patient data, and thereby enable the development of target-specific therapies and clinically differentiated products in the rare disease area. This was to be achieved through collaboration with expert physicians, patient organisations and registry development experts. In Australia, there presently is no national strategy in place for people living with rare diseases. It is envisaged that one element of a national rare disease plan should include patient registries and the collection of relevant data to benefit all stakeholders involved in developing and using innovative treatments. The creation of an IRDR will serve as a key instrument for building and empowering rare disease patient communities. Such a registry would enable all rare disease stakeholders to achieve their different but complementary goals aimed at augmenting knowledge and developing new therapeutic options for the future. As a priority, it was proposed that the Gaucher registry (GR) would be developed and deployed as the first IRDR.

Several existing drug registries collect data on the efficacy, treatment outcomes or toxicity of the three available enzyme replacement therapies; the International Collaborative Gaucher Group (ICGG) Gaucher Registry (imiglucerase, supported by Genzyme, a Sanofi Company) [4], [5], the Gaucher Disease Enzyme Replacement Therapy Registry (taliglucerase alfa, supported by Pfizer) [20], and the Gaucher Observational Study (GOS, velaglucerase alfa, Shire Human Genetic Therapies). Additionally, several national registries also operate in Spain [7], [8], Brazil [9], and France [6].

For Australian treated patients with Gaucher disease however, manual spreadsheets were kept for all patients, with only one industry-managed registry available for some data entry (the ICGG Gaucher Registry). This is not an ideal situation, especially since a single pharmaceutical company held the database, even though patients were subsequently treated with multiple drugs from different companies. The GR described in this manuscript therefore seeks to include all Australian patients with Gaucher disease, irrespective of treatment status and independent of treatment type.

A component of the Pathfinder Award provided for the development of an IRDR that would allow for an increase in knowledge on rare diseases by pooling data from clinical and epidemiological research and real-life drug use in communities. This is of particular importance in Australia, a country that is sparsely populated and has a unique ethnic diversity with 3% of the population Aboriginal and/or Torres Strait Islander [10], and 28% first generation migrants from over 200 countries [11]. A well implemented IRDR will increase opportunities not only for rare disease treatments to be developed but also to enable independent post-marketing review of clinical efficacy and safety. Consistent long-term collection of patient data will help create standards of care (including a minimum set of common data elements) that in turn can dramatically improve patient outcomes and life expectancy, even in the absence of new therapies. The IRDR would provide unique insight into the detail of a country's health, social services planning and healthcare organisation.

A recent article by Hollak et al. [2] supported the call for independent registries in Europe. The authors argue that ideally: registries are disease-centred with data captured from pivotal, extended trials, natural histories and quality of life studies; they are not drug-centred; appropriate governance and patient data analysis is independent of corporate influence; mandatory data capture for all doctors treating patients across Europe; and the launch of registries early in the development process of orphan drugs. Thus, there is alignment between the motivation of the Wellcome Trust Award and the requirements outlined in the article by Hollak et al. [2]

Here we define what we believe are core features of an IRDR and describe a registry framework that meets these needs, the Rare Disease Registry Framework (RDRF). The RDRF has been tailored to Gaucher disease as the model rare disease, for which therapies are available from a number of drug companies.

## 2 Materials and methods (registry system overview)

The open source RDRF allows the efficient deployment of web-based registries that can be modified dynamically as registry requirements evolve [12], [13], [14], [15], [16]. The RDRF employs a modular approach to registry design that empowers registry administrators to easily configure registries without software developer effort, by allowing users to dynamically create all data elements (DEs), sections and forms that define a patient registry and to share DEs across registries. Registries are described in a computer-readable text file, which allows a registry definition to be imported/exported, versioned, and stored in a shared accessible environment. Multiple registries can also be deployed on a single site, and patients can be defined once, yet belong to multiple registries.

The RDRF allows multiple levels of access, through the dynamic configuration of working groups, user groups, and permissions. User groups may include clinical, genetic, curator and administration users, which can be configured to have access to different forms and certain registry functionality. Users are assigned access to working groups that may be a clinic, state, or country jurisdiction. Users are only able to access the data of patients who are assigned to the working groups for which they have access permission, enabling efficient collaboration for data entry across jurisdictions (nationally, or internationally).

The RDRF not only facilitates the effective capture, storage, management and access of patient information, it is also interoperable with other systems from which it can capture, import and store data, thereby integrating patient details with clinical data and results [14]. The ability to re-use DEs across multiple registries greatly assists in the standardization of data capture, allowing for effective data sharing for research purposes.

The RDRF takes a conceptual approach to the design and development of patient registries to ensure access, security, privacy, and to meet the need for harmonisation across multiple clinical sites in a given country, or internationally. The RDRF also fulfills the key criteria required for sustainable registry development, such as open-source, modular design, web-based, provision of an application programming interface (API), and interoperability [13], [17], [18], [19].

We engaged with an established rare disease community for Gaucher disease to determine the system requirements, methods of data capture, consent, and reporting, and tailored the RDRF to meet these requirements for the deployment of a Gaucher disease registry.

## 3 Results and discussion

Through detailed interactions with clinicians, patient advocates and industry, we identified a significant number of challenges that needed to be addressed to establish IRDRs. These included: long-term sustainability once a registry is deployed; development and ongoing registry enhancements; dynamic consent that can be modified once a registry is deployed; standards for data elements, sections, and forms that can be shared and which are not hard-coded into the registry; and data that are not only time-stamped but also captured within a time-stamped context.

The Rare Disease Registry Framework (RDRF) described in this manuscript has an extensive array of features to address these challenges, as listed in Supplementary Table S1. The key functionality focus of the RDRF was to develop a generic IRDR using Gaucher disease as the specific model, since there are already a number of therapies available for this condition, with only one international registry open to Australian patients (the ICGG Gaucher Registry) operating within a single drug company (Genzyme, a Sanofi Company). The framework for this registry has been specifically designed for functionality and customisation to be available at the end-user level rather than at the system development level. A number of these key features are highlighted in the following sections.

### 3.1 Context

**Fig. 1:**
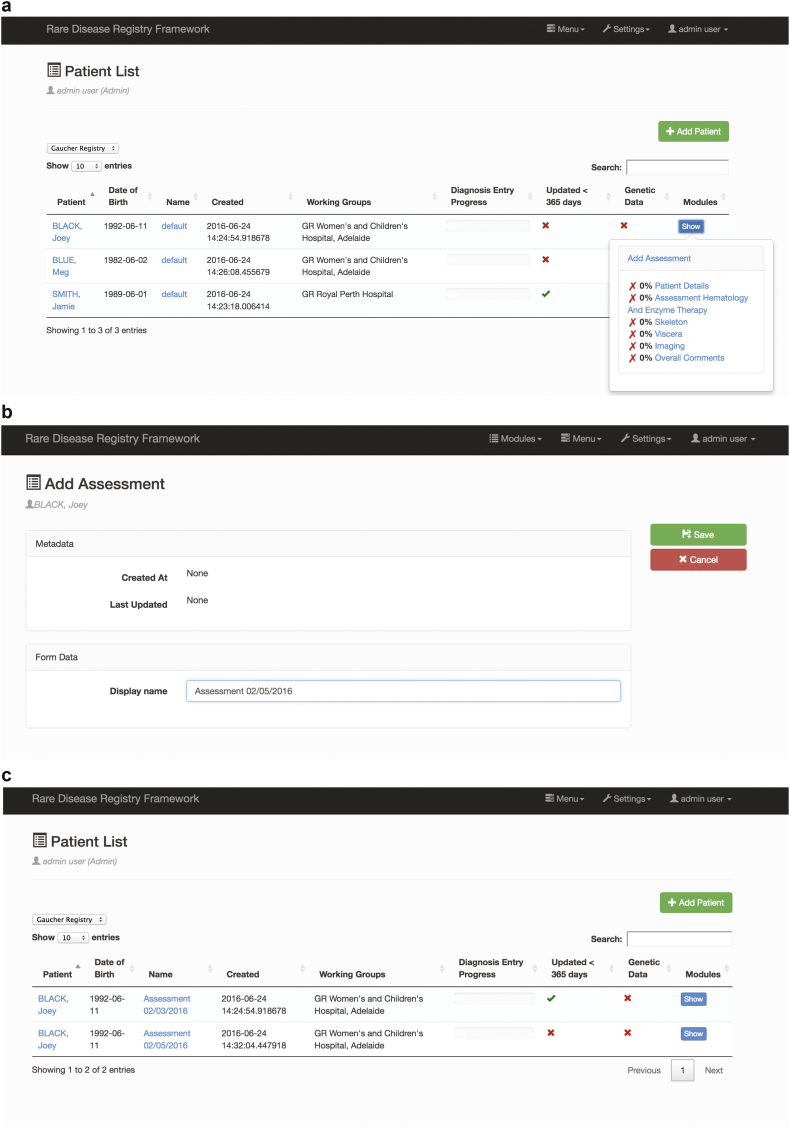
Details of context. a) The ‘Patient Listing’ is where patients are viewed, and new patients are added by clicking the green ‘add patient’ button. The implementation of ‘context’ allows new Assessments to be added for each patient, so all data entered into the registry may be viewed (previously only the latest data could be viewed); b) New Assessments are created as needed. The Demographics and Consent Modules are defined once when the patient is first created, and the collection of Forms are created with each new Assessment; c) New patient listing page for each individual patient showing all ‘Assessments’ and progress details.

A key requirement for a GR was to enable the capture of data in a given context. We recognised a subtle but important distinction between time-stamping data entry in a data field versus time-stamping data entry according to an assessment date. We refer to the latter form of time-stamping as a *context*. For Gaucher Disease the context is ‘Assessment date’. Without this functionality it would be difficult for a registry system to support post-authorisation assessment. The RDRF can now support both forms of time-stamping for more general application to other diseases. Naturally, analytics and visualisation of patient data can be conducted more meaningfully at a *context* level. Fig. 1 provides details of context.

### 3.2 Customisable consent

**Fig. 2:**
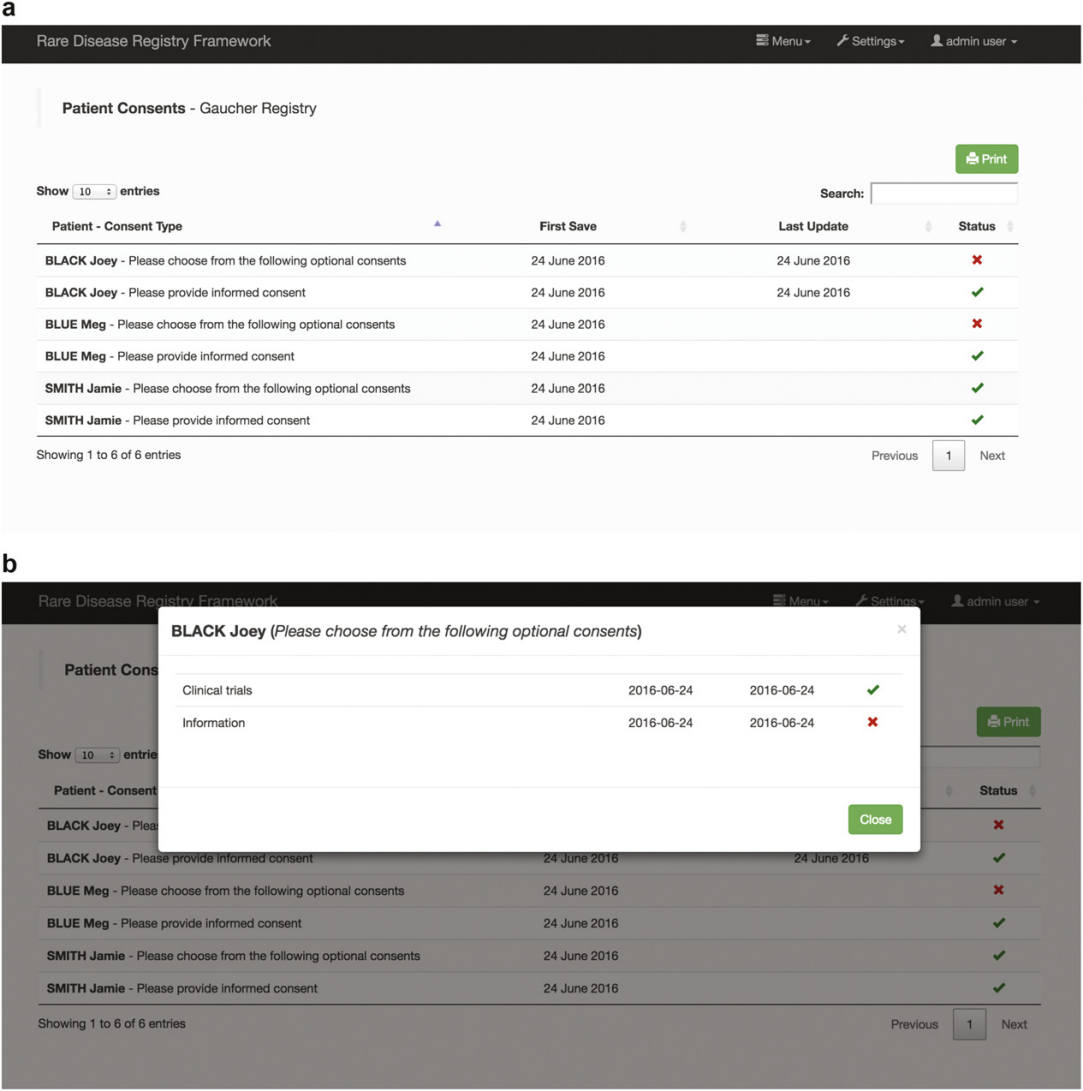
Configurable and customisable consent. a) Consent is now easily customised, with validation and applicability rules available. Time-stamping of when consent is provided in a searchable table of consent sections. This assists Data Curators to identify patients who have not provided informed consent; b) Further information on individual consent questions can be obtained by clicking on the patient name.

The RDRF has the ability to configure and customise consent (Fig. 2). From extensive stakeholder engagement it became clear that consent for a given disease registry is not static and should not be hard-coded into the registry development. Consent requirements can change, and individuals may choose to ‘modify’ their consent over time. When initially designing the consent model, in the RDRF an end-user administering the patient registry is readily able to modify the consent and can choose to define the required consent validation and application rules. For example, consent criteria must all be ‘checked’ or only a selected ‘mandatory’ number agreed upon. Thus functionality/access to other functions within a given registry will be determined according to these rules. Having a digitised patient accessible and modifiable consent will facilitate data-sharing with other researchers to enhance collaborative investigations on rare disease cohorts.

### 3.3 Registry description language

The RDRF employs a description language [14] for all registry definitions. A Registry Mark-up Language (RML) can define a complete registry, a registry form, section, data element, permissible value and permissible value group. Use of the RML means that a standard registry definition can be shared. Similarly, definitions for forms, sections and data elements can be shared. Note that sharing a registry RML is not sharing the data contained within a given registry. The RML is located in a shared online environment and can be categorised according to any given ontology for both registries and data elements. For example, NINDS define ‘common’ data elements and disease-specific data elements [21].

### 3.4 Security and multi-level access

From both application level and operational levels the RDRF supports an array of security options. From an application level perspective, the RDRF is built on top of a technology framework that has long-term support [22]. This framework itself provides distinct levels of built-in security including: Secure Socket Layer security encrypting all web traffic to and from the application; Cross-Site Request Forgery checking that is a method of ensuring that falsification of form submissions is near impossible; and login restrictions of all “views”. In addition, the RDRF itself includes a fully configurable permissions layer (role-based security model) that restricts the visibility of forms and fields to specified user groups. Furthermore, the RDRF stores identifying patient contact/demographic data in a database that is totally distinct from any clinical/genetic data.

From an operational-level perspective, any deployment of a registry will need to address operational security. This is security relating to the environment in which the software runs, and cannot be addressed by the software itself. The registry framework stores data in PostgreSQL and MongoDB. The databases using these systems are encrypted, which ensures that data are protected if the storage hardware is (for example) stolen, reused, or returned to the manufacturer to address a fault. In this sense, storage includes all physical media that are used to store registry data, including the volumes used by the database software, the volume on which the front end is installed, and any volumes used for operating system “swap” space. Communication between the web interface and the databases is encrypted to guard against confidential data being intercepted “in transit”.

In terms of physical security, workstations including laptops used to access the registry should require user authorisation, be subject to appropriate security policies, and have appropriate security software installed. On any workstation on which reports may be downloaded from the registry and stored, whole-disk encryption should be implemented on the device to guard against the risk of data exposure through theft or accidental loss.

### 3.5 Reporting engine

**Fig. 3:**
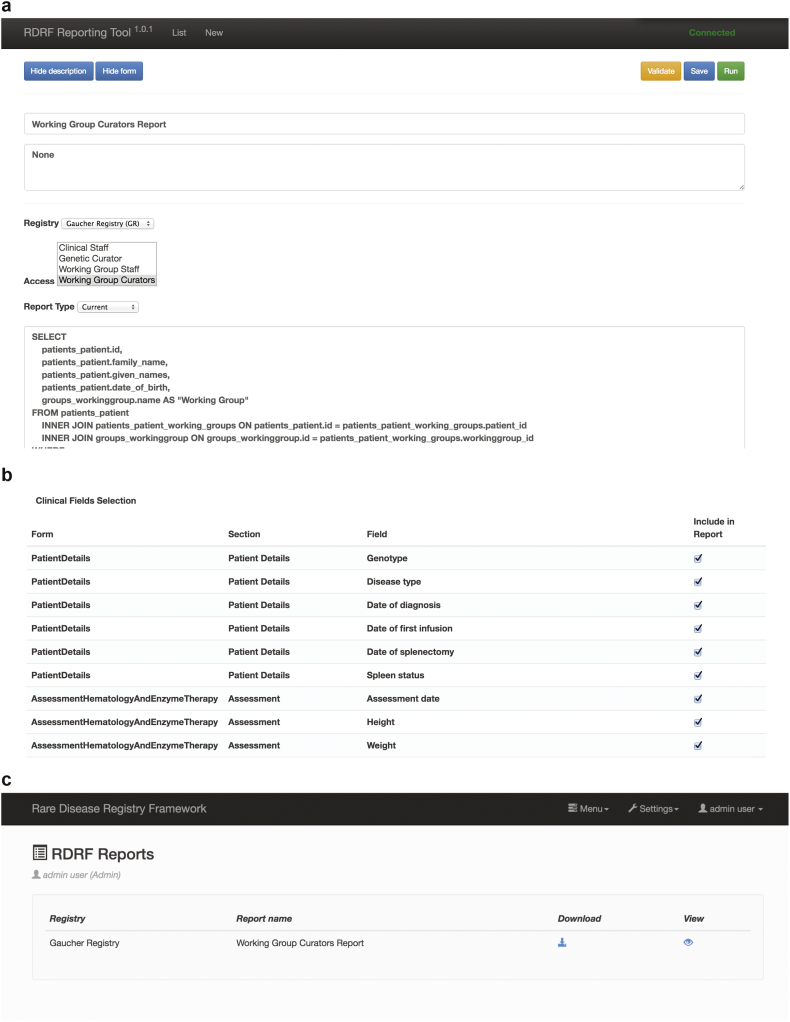
Reporting engine. a) Reports are customised through the reporting tool—access can be configured to certain user groups; b) Data Elements can be easily chosen for inclusion in the report by clicking a check box; c) Once a report is configured, it may then be saved and viewed or downloaded—reports are generated at download with the latest registry data.

The RDRF also supports a reporting engine. A user with administration privileges is able to customise the data contained within the registry to generate a report, which can be configured to be accessed by certain user groups (Fig. 3). This report template can be saved and reused as required. The reporting engine also enables various types of reports to be generated, such as ‘current’ or ‘longitudinal’. While SQL queries are used to select and aggregate demographic data as required, simple check boxes for each DE contained in the registry (check for inclusion in the report) also enable ease of report construction as they minimise the use of SQL queries.

### 3.6 Interoperability

A key feature for any registry is its ability to communicate with other registries in a programmatic known as *interoperability*. The RDRF has implemented an application programming interface (API) to achieve this outcome. This API can be used to interrogate other systems, e.g. other registries or allied systems, such as biobanks, via their APIs. Alternatively, if permissions are provided other systems can interrogate a given registry implemented using the RDRF.

### 3.7 Sustainability

From our perspective, addressing an IRDR's sustainability is paramount. In this regard we consider four important dimensions to an IRDR sustainability: utility, effectiveness, efficiency and agility. While the first three dimensions are broadly acknowledged in achieving sustainable solutions in general, we see the fourth dimension as critical when considering registries. We have previously described patient registries as non-static entities. Registry requirements, their purpose, functionality, and access privileges evolve over time. For instance, consent, either from a policy or individual's permission might change, new data fields reporting new phenotypic measures or refinement of existing data elements must be catered for, or a registry might initially be intended as a contact registry with functionality subsequently incorporated to convert it to a clinical registry [13].

A sustainable registry must be interoperable with other registries and to allied systems, such as biobanks and clinical information management applications, be fit for purpose, yet customisable for end-users with minimal requirements of software developers. This last point in particular, is crucial in the facilitation of a sustainable registry as it dramatically reduces registry maintenance costs without limiting the flexibility of the system to customise registries. The registry described in this paper has been developed to facilitate sustainability and in this regard it meets each of the criteria listed above.

## 4 Conclusions

The RDRF has been developed with a focus on sustainability. A sustainable registry must be able to interoperate efficiently with other systems via an API. Such systems include other patient registries, biobanks and clinical information management systems. A sustainable registry must also be open source, fit for purpose, customisable for end-users, and by end-users, with minimal involvement of software developers.

The RDRF has an extensive array of features. From our end-user engagement we have recognised that a number of features that might be considered bespoke by one rare disease community can, in fact, be generalised so that these features can become available to other rare disease communities. The advantage of the RDRF reported in this paper is that it will allow, and include, the incorporation of data directly reported by patients along with clinical information reported by healthcare professionals. This will improve the robustness, comprehensiveness and quality of the data entered. Furthermore, modular additions, or adjustments to the fields, do not require high level software input.

The IRDR project will serve as a key instrument for building and empowering new and existing patient communities, augmenting knowledge, patient care and developing new therapeutic strategies for the future. It will facilitate a collaborative approach to the collection of company independent post-marketing clinical outcome data that will rationalise best practice utilisation of orphan drugs. The IRDR will thus improve decision-making of health care providers at a national level, facilitating cost-effective programs for the subsidised management of these extremely expensive drugs.

The following is the supplementary data related to this article.

Supplementary Table S1 Key features of the Rare Disease Registry Framework (RDRF). Supplementary Table S1

## Funding

This work was supported by the Wellcome Trust Pathfinder Award [REF 104746] with financial contribution to this award by Shire Australia Pty. Ltd. The authors gratefully acknowledge the combined support-in-part funding for this work. This includes Australian National Health and Medical Research Council [APP634485, APP1055319] and the EU FP7 Project [HEALTH.2012.2.1.1-1-C]: RD Connect: An integrated platform connecting databases, registries, biobanks and clinical bioinformatics for rare disease research.
