# Supplementary material for: Design of a framework for the deployment of collaborative independent rare disease-centric registries: Gaucher disease registry model
Source: Blood Cells Mol Dis. 2018 Feb;68:232–8. doi: 10.1016/j.bcmd.2017.01.013 (PMC5729019; doi:10.1016/j.bcmd.2017.01.013)
Supplement: Supplementary Table S1 — Key features of the Rare Disease Registry Framework (RDRF). [file mmc1.docx]

**Supplementary Table S1: Key features of the Rare Disease Registry Framework (RDRF)**

| **FEATURE** | **DESCRIPTION** |
| --- | --- |
| **Data Elements and Permissible Values** | |
| - Dynamic Creation of Data Elements | Users of the RDRF (typically assigned administrators) have the ability to add new Data Elements (DEs). DEs can be reused. |
| - Support for user defined Derived Data Elements | A Derived Data Element with a calculated designation can dynamically generate a value based on the values of other defined Data Elements or data object model. |
| - Data Element support for various “abstract data types” | Framework supports: String (allows pattern matching/restrictions to be imposed); integer (with max/min); range (list/permissible values); calculated (functions); file (upload/download); float (real/decimal numbers); alphanumeric; Boolean (true/false presented as a check box), date. |
| - Dynamic creation of Permissible Values and Permissible Value Groups | Permissible Values are assigned to a Permissible Value Group, used to denote a list for range data types. Permissible Value Groups may be reused in different DEs. |
| - Widgets can be assigned dynamically to Data Element fields | Widgets can be selected dynamically (at runtime). This allows different display components (e.g. Date fields) to be chosen at run time. For instance, a Date Picker presents a calendar widget. |
| **Sections, Forms and Registries** | |
| - Dynamic creation of Sections | Sections consist of a collection of DEs, and can be reused. |
| - Dynamic multiplicity of fields for some form sections | A form section marked as ”multiple” allows its fields to be dynamically added or removed en block (e.g. a multiple contacts section could list contact name, email, relationship as three fields - by marking the section as multiple the framework adds an add/remove button to the page which allows multiple contacts to be added). |
| - Dynamic Creation of Registry Forms | Forms consist of a collection of Sections. Each Registry is made up of a collection of Forms. |
| - Progress Indicator | DEs on a Form can be selected to contribute to ‘progress completion’, which is represented by a progress indicator bar on the Form itself. On the Patient Listing page, green ticks and red crosses indicate if data has been entered under ‘Show Modules’ |
| - Dynamic creation of a Registry | More than one Registry per web site is possible. |
| - Dynamic creation of Questionnaire page for a Registry | Nominating a form as a questionnaire exposes the form on a public URL. The data captured by the form is stored as a “questionnaire response” which when approved by a curator, creates the patient record and also updates the clinical data record for the new or existing patient. |
| - Questionnaire validation | Moderated workflow for questionnaire submission. |
| - Export Registry Definition File | A Registry is defined in Registry Definition File. This file can be exported from one RDRF installation to another (YAML format). |
| - Import Registry Definition File | Enabling another RDRF installation to duplicate a complete Registry (YAML format). |
| **Customisable roles, access permissions, views and consent** | |
| - Customisable roles | Each workflow in RDRF is intended to be performed by a user with a distinct role. Current roles in RDRF are admin, working group curator, working group staff, clinical staff, genetic staff, genetic curator, parents and patients. This is customisable in ‘Users’. |
| - Dynamic creation of Users | Users can be created and assigned to a specific role (e.g. working group curator, clinical staff), assigned a username and password, and restricted to certain working groups and/or registries. |
| - Access permissions can be modified dynamically | Access permissions can be customised for each role in ‘Groups’. |
| - Access permissions of Forms can be set | Forms can be restricted to be viewed and edited by certain Users (e.g. Clinical Data Form can be restricted to be viewed only by working group curators and clinical staff in the Form definition). |
| - Dynamic creation of working groups | Working groups can be created dynamically (e.g. a hospital, or state). Users can be assigned to multiple working groups. |
| - Restriction of Demographics Fields | View of demographics fields can be restricted by Registry and User, and designated as read only or hidden. |
| - Dynamic Consent, Validation and Applicability | Multiple levels of consent can be defined for each registry, with validation rules applied (e.g. must check one of two mandatory consent options for a patient to be created). Applicability conditions may also be applied (e.g. different sets of consents are displayed for patients of different age groups). |
| - Configurable views for the Patient Listing | View of the Patient Listing can be configured for different users by applying permissions to show the following columns:   - Data modules - Date of birth - Patient Name - Working groups - Progress indicator - Genetic Data (notes if data has been entered by ticks and crosses) - Updated last 365 days (notes if data has been entered by ticks and crosses) |
| **Context** | |
| - Context | Time-stamping of data entry according to an ‘Assessment date’. Groups of Forms or individual Forms can be designated to have a context. |
| **Patient Registration** | |
| - Patient registration and log-in | Patient Registration can be configured. Patients register through an online Registration Form and have the ability to log into the registry and enter their own data. |
| **Automated Notifications** | |
| - Email notifications | Email templates and email notifications can be configured and sent from the system. |
| **Genes, Laboratories and Doctors Modules** | |
| - Genes Module | A list of Genes can be utilised in a GeneLookupWidget. |
| - Laboratories Module | A list of testing Laboratories can be utilised in a LaboratoryLookupWidget. |
| - Doctors Module | A list of Doctors can be selected in the ‘Patient Doctor’ sections in Patient Demographics. |
| **Security/Reporting/API features** | |
| - IP address restrictions | Within the RDRF it is possible to define external IP (Internet Protocol) address to ban or allow user(s) from accessing registries defined within the RDRF. |
| - User login attempts auditing | Audit trails of all user login attempts. |
| - Reports of registry data | Reports can be built by an admin user using the ‘Explorer’ tool, and downloaded in .csv format. SQL queries are used for demographic data, while simple check boxes allow DEs of interest to be included in the report. Reports may be designed to include current or longitudinal data, and may also be configured in spreadsheet format over multiple spreadsheets. |
| - Exposed REST web service | Allows for patient data to be updated/retrieved by any client that can create HTTP requests. |
| - API | used to interrogate/connect to other systems |
| **Other features** | |
| - Molecular sequence | HGVS annotations can be captured. |
| - Longitudinal data snapshots | Storage of longitudinal snapshots of data. Can also be viewed directly on reports through ‘view previous results’. |
| - Patients in multiple registries | Within the RDRF, patients can be in one or more registries without the need of duplicating patient information. |
| - One RDRF = Multiple registries | Multiple registries can be managed in one installation. |
| - Open source and RDRF Deployment | RDRF is open source. Source code is available at https://github.com/muccg/rdrf |
| - Registry landing page | It is possible to create a customisable landing page for each registry. |
| - Demo available | <https://rdrf.ccgapps.com.au/demo/> (username and log in: admin \| admin; curator \| curator; clinical \| clinical; genetic \| genetic) |
| - Project website | https://muccg.github.io/rdrf/ |
| - User documentation | https://muccg.github.io/rdrf/docs/ |
